# Variation in Growth, Morphology, and Fungicide Sensitivity Among *Monilinia* Species from South Tyrol’s Alpine Orchards

**DOI:** 10.3390/jof11100690

**Published:** 2025-09-23

**Authors:** Melanie M. Pagano, Sabine Oettl, Evi Deltedesco, Youry Pii, Urban Spitaler

**Affiliations:** 1 Institute for Plant Health, Laimburg Research Centre, Laimburg 6, 39040 Auer (Ora), Italy; 2 Faculty of Agricultural, Environmental and Food Sciences, Free University of Bozen-Bolzano, Piazza Università 5, 39100 Bolzano, Italy

**Keywords:** brown rot, *Monilinia* species, fungicide, conidia, sporulation

## Abstract

Brown rot, caused by *Monilinia* species, is a major disease affecting stone and pome fruits. The most relevant species are *M. fructigena*, *M. laxa*, *M. polystroma*, and *M. fructicola*. These four species exhibit morphological differences, but comparative data on these traits remain limited. In European integrated fruit production, pre-harvest control of brown rot mainly relies on the fungicides cyprodinil, boscalid, and tebuconazole. Given the coexistence of multiple *Monilinia* species, understanding differences in fungicide sensitivity is crucial for optimizing control strategies. In this study, mycelial growth, colony and conidial morphology, and sporulation capacity on potato dextrose agar (PDA), tomato sauce agar (TSA), and apple fruit were investigated. Fungicide sensitivity was assessed by measuring mycelial growth on apples following treatment with the three active ingredients. Tebuconazole was the most effective fungicide, particularly against *M. laxa* and *M. polystroma*, while cyprodinil and boscalid were less effective. These results highlight the need for species-specific fungicide strategies, *Monilinia* populations, and the effectiveness of disease control under various environmental conditions. All *Monilinia* species sporulated successfully on TSA, underscoring its suitability for sporulation studies. Conidial size varied significantly among species; nevertheless, overlapping sizes prevent reliable species or strain discrimination based on this trait alone.

## 1. Introduction

Brown rot, caused by *Monilinia* species, is one of the most important diseases in stone fruit orchards, both pre- and postharvest. *Monilinia* species are a significant threat to stone and pome fruit crops, particularly in temperate climates. The most important pathogens include *Monilinia fructigena* Honey, *Monilinia laxa* Honey, *Monilinia polystroma* L.M. Kohn, and *Monilinia fructicola* Honey [1]. All *Monilinia* species affect the flowers and twigs of stone fruit, causing blossom blight and cankers [2,3,4]. Additionally, the symptoms of brown rot appear on the stone fruit. Initially, small, superficial, brown circular spots are observed, which expand in diameter until they cover the entire fruit [5]. The fungus overwinters primarily in mummified fruits, which either remain on the trees or fall to the ground [6,7]. In pome fruit cultivation, *Monilinia* is mainly recognized as an important post-harvest pathogen [4,6]. However, brownish dieback has also been observed on the leaf petioles, laminas, and on small fruits and fruit pedicels of apple [8]. Since brown rot symptoms are caused by different closely related species, morphological characteristics of individual strains are crucial for distinguishing *Monilinia* species and understanding the consistency between strains.

Management of brown rot relies on cultural practices, chemical treatments, biological control, and, finally, physical treatments to reduce or eliminate harvest losses. The highest efficacy is achieved with chemical control, particularly through preventive applications [9]. In Europe, chemical post-harvest treatments against *Monilinia* are not authorized [10]. As a result, brown rot infection after harvest ranges up to 30%, with peaks up to 50% [11,12].

Three active ingredients are commonly applied in the orchards: cyprodinil, boscalid, and tebuconazole. Cyprodinil operates through a systemic mode of action (MoA) as a single-site methionine inhibitor, targeting the enzyme cysteine β-lyase. Based on its MoA, the Fungicide Resistance Action Committee (FRAC) classifies it in Group 9 as an anilinopyrimidine (AP) [13]. Boscalid acts as a contact fungicide with a likely multi-site MoA. It inhibits the ubiquinone binding site QP, disrupting the electron flow to Complex III and coenzyme Q, causing oxidative stress, blocking respiration, and resulting in ATP depletion [13,14,15]. Boscalid belongs to FRAC Group 7, succinate dehydrogenase inhibitors (SDHI) [13]. Tebuconazole is a systemic fungicide with a single-site MoA that inhibits demethylation and other processes in sterol biosynthesis. Its effects include mycelial growth inhibition and suppression of sporulation [16,17]. Tebuconazole is classified in FRAC Group 3 as a demethylation inhibitor (DMI) [13]. Demethylation refers to the chemical process of removing a methyl group (CH3) from a molecule. To assess the damage potential of different species, it is essential to know the species-specific sensitivity to fungicides.

Recent studies on *Monilinia* species in stone and pome fruit have identified all four species in South Tyrol (Italy), where the present study was conducted [18,19,20]. Research on *Monilinia* strains from this region is particularly valuable because South Tyrol is an alpine area where stone fruit cultivation occurs mainly in cooler mountain regions. This environment may favor brown rot caused by *M. laxa* rather than *M. fructicola*, as the optimal growing temperature for *M. fructicola* is 26 °C, which is significantly higher than the 20 °C and 23 °C optimal for *M. fructigena* and *M. laxa*, respectively [1,21].

Despite several studies on pathogenicity, limited information exists regarding the morphological, phylogenetic, and fungicide-resistance profiles of various *Monilinia* species. In particular, the species *M. polystroma* lacks detailed information [1,22,23,24]. Therefore, additional information on this species would be valuable for distinguishing *M. polystroma* from other species.

Hence, the present study aims to investigate in detail the morphological and phylogenetic characteristics of *M. fructigena*, *M. laxa*, and *M. polystroma*, as well as a reference isolate of *M. fructicola*. Furthermore, the sensitivity of these species to the active substances cyprodinil, boscalid, and tebuconazole was assessed to determine if species-specific tolerance is present. Particular attention was given to *M. polystroma* due to the limited available information on this species.

## 2. Materials and Methods

### 2.1. Fungi Isolates and Experimental Conditions

The used strains were isolated from fruit mummies and infected fruit sampled in 2020 and 2021 in South Tyrol [18,19,20]. To obtain single *Monilinia* strains, single-spore isolates were prepared from symptomatic fruit. Spores were picked with a needle under sterile conditions using a stereo microscope and transferred to Petri dishes (90 mm diameter) containing potato dextrose agar (PDA; 4 g L^−1^ potato starch, 20 g L^−1^ dextrose, 15 g L^−1^ agar, pH 5.2; Difco™, Bordeaux, France). Details on single strains are available in Table 1. Incubation and all experiments were performed in a climatic chamber at 20 ± 1 °C, 40% RH, and under a 12:12 h L:D (light:dark) photoperiod. Light was generated by an LED Osram Fluora L 36W/77 (Fluora Leuchten Lichtmanufaktur AG, Flawil, Switzerland), providing white light.

### 2.2. Phylogeny

Total DNA was extracted from monoconidial culture grown on PDA using the DNeasy^®^ Plant Mini Kit (Qiagen GmbH, Hilden, Germany) according to the manufacturer’s instructions with minor modifications: the fungal material was disrupted by adding 400 μL of AP1 lysis buffer and three 3 mm tungsten carbide beads (Qiagen) in a mixer mill (MM 400; Retsch GmbH, Haan, Germany) for 3 min at 30 beats per s. No RNase was added. The DNA was eluted two times with 50 μL nuclease-free water. The DNA concentration of the samples was quantified using a NanoDrop 1000 spectrophotometer (Thermo Fisher Scientific, Wilmington, NC, USA) and stored at −20 °C until use [19].

The internal transcribed spacer (ITS) region was amplified using the universal primers ITS_5_ and ITS_4_ [25]. For the PCR reactions, 30 µL reaction mixture was used: 1 × Phusion HF Buffer, 0.2 mM dNTPs, 0.4 µM of each primer, 0.02 U µL^−1^ Phusion^TM^ High-Fidelity DNA polymerase (Thermo Fisher Scientific Inc., Waltham, MA, USA), and 3 µL DNA template. The cycling conditions consisted of an initial denaturation at 95 °C for 2 min, followed by 35 cycles of denaturation at 95 °C for 30 s, annealing at 56 °C for 30 s, and extension at 72 °C for 45 s, with a final extension at 72 °C for 5 min.

PCR products were run on a 1.5% agarose gel, stained with GelRed^®^ Nucleic Acid Stain (Merck KGaA, Darmstadt, Germany), and viewed on a ChemiDoc^TM^ Imaging System with Image Lab^TM^ Software version 5.2.1 (Universal Hood III, Bio-Rad Laboratories Inc., Hercules, CA, USA). Successfully amplified products were sent for Sanger sequencing to LGC Genomics GmbH (Berlin, Germany). Raw sequence data were checked manually and trimmed using Geneious v.11.1.5 (Biomatters Ltd., Auckland, New Zealand). Multiple sequence alignment was performed using the ClustalW algorithm to prepare the sequences for phylogenetic analysis. Subsequent phylogenetic analysis was performed, including one with MEGA v. 12.0.11 [26]. A model test to identify the lowest Bayesian information criterion (BIC) score was performed, and the Kimura 2-parameter (K2) model was applied for maximum likelihood (ML) tree reconstruction. For bootstrap analysis, 1000 replicates above 85% were performed to estimate node support.

### 2.3. Growth Rate and Colony Morphology on PDA

To characterize the *Monilinia* strains, in vitro tests were conducted on PDA (39 g L^−1^). Petri dishes (90 mm diameter) were inoculated with mycelial plugs from a PDA plate, mycelial side down, and sealed with parafilm. Radial growth (mm) and colony morphology were assessed at 6, 10, 15, 20, and 27 days post-inoculation (dpi). Measurements were taken on the back of the plate at the edge of the inoculated area, and the mean value per strain was calculated from four radial measurements. Five replicates were performed (*n* = 5).

### 2.4. Mycelial Growth Rate and Morphology on Apple

Differences in the characterization of *Monilinia* species strains were evaluated through *in planta* tests on apples of the Golden Delicious cultivar from organic production after five months of storage. Apples were surface sterilized using cotton swabs soaked in 75% ethanol. A 3 mm deep hole was punched into each fruit with a 7.8 mm diameter cork borer and a mycelial plug, matching the hole’s diameter and taken from the colony periphery, was placed inside with the mycelial side facing the pulp. Sterile PDA plugs were used as controls. Apples were placed on the halves of 6 cm diameter Petri dishes and incubated in sterile, transparent plastic boxes (22 × 12 × 33 cm; Gió Style, Rome, Italy) containing 100 mL of sterile water-soaked bibulous paper. The box lids were left slightly ajar to allow air circulation. Five apples per strain were placed in each box. The relative humidity inside the boxes was nearly 100%. Radial fungal growth on the apples was monitored until the fruit was fully colonized. Measurements were taken at 7, 9, 14, 16, 21, and 23 dpi. Fungal coverage was measured at four positions from the edge of the inoculated hole to the fungal margin using a ruler, and the growth rate was calculated. The mean of these four measurements was used for statistical analysis, with each apple serving as a replicate (*n* = 5).

### 2.5. Conidia Production and Characterization

Sporulation and conidial size were assessed using the same apples described above. Conidia from inoculated apples were collected and placed on Fuchs–Rosenthal chambers for evaluation. Incubation period of the apples ranged from 9 to 21 dpi. If no sporulation occurred on apple, it was induced on tomato sauce agar (TSA; 227 g tomato sauce (Casar Srl, Serramanna, Italy), 20 g agar (Merck, Darmstadt, Germany), 750 mL deionized water) following the method of [27]. Dishes were inoculated with mycelial plugs taken from the periphery of PDA-grown cultures, with two technical replicates.

Sporulating samples were scraped using sterile inoculating loops, and conidia were suspended in tap water on Fuchs–Rosenthal chambers. Microscopic analysis was conducted at 200× and 400× total magnification (20× or 40× objective plus 10× ocular). The length and width of 50 conidia per sample were measured (*n* = 50).

### 2.6. Fungicide Screening on Apples

Three plant protection products, each authorized for use in integrated pest management (IPM) in Italy (Table 2), were tested for their efficacy. Fungicide sensitivity was evaluated under *in planta* conditions, following the methodology described by Chen et al. (2013) [27]. Apples were washed twice with tap water, sterilized using cotton swabs soaked in 75% ethanol, and then left to air dry. Application rates for the active substances were based on the manufacturers’ recommended field doses for controlling *Monilinia* species (http://www.fitosanitari.salute.gov.it, accessed on 15 March 2025). Approximately 1.5 mL of each fungicide was sprayed onto the apples using a hand mister, ensuring coverage to the point of runoff. Sterile deionized water was used as a control treatment. Each treatment included five apples (*n* = 5). Inoculation and evaluation procedures were conducted as described above. Growth inhibition was calculated as the coverage in the fungicide treatment relative to the mean coverage in the control. A 100% inhibitory effect indicates complete growth suppression, whereas 0% inhibition corresponds to growth equal to or greater than that of the control.

### 2.7. Statistical Analyses

All statistical analyses were performed using R version 4.3.2 (R Foundation for Statistical Computing). Mycelial growth on PDA and apples, as well as fungicide efficacy, was evaluated using linear mixed-effects models (LMMs) fitted via restricted maximum likelihood (REML) with the lme4 package [28]. Species or strain were included as fixed effects, while dpi was treated as a random effect. Data from different species were analyzed separately to assess the effect of individual strains. Model selection was based on Akaike Information Criterion (AIC) values, and residuals were examined to verify error distribution assumptions. Pairwise comparisons of treatments were conducted using Tukey’s test. The lmerTest and multcomp packages were used for statistical inference, and figures were generated using ggplot2 [29,30,31].

## 3. Results

### 3.1. Phylogeny

**Figure 1:**
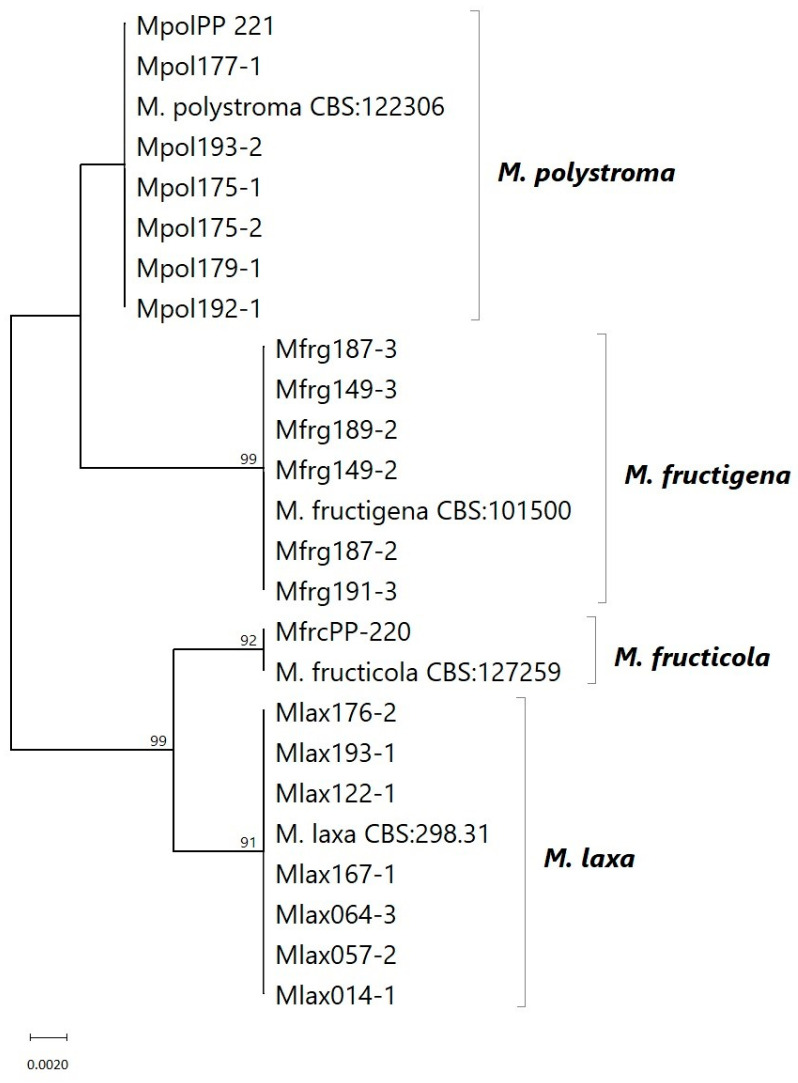
Maximum likelihood (ML) tree based on ITS sequences from *Monilinia* strains collected in different locations in South Tyrol, Italy. Sequences from NCBI GenBank were used as references. Bootstrap support values (1000 replicates) above 85% are shown at the branches.

The identity of the four *Monilinia* species was confirmed through BLASTN analysis of the obtained ITS sequences against reference sequences in the NCBI GenBank database. The isolates clustered with the reference sequences of *M. fructicola* CBS 127259 (MH864497), *M. fructigena* CBS 101500 (MH862738), *M. laxa* CBS 298.31 (MH855219), and *M. polystroma* CBS 122306 (MH863200) [32]. No genetic differences were observed between strains originating from different geographic locations or host fruits within the same *Monilinia* species (Figure 1).

### 3.2. Growth Rate and Colony Morphology on PDA

**Figure 2:**
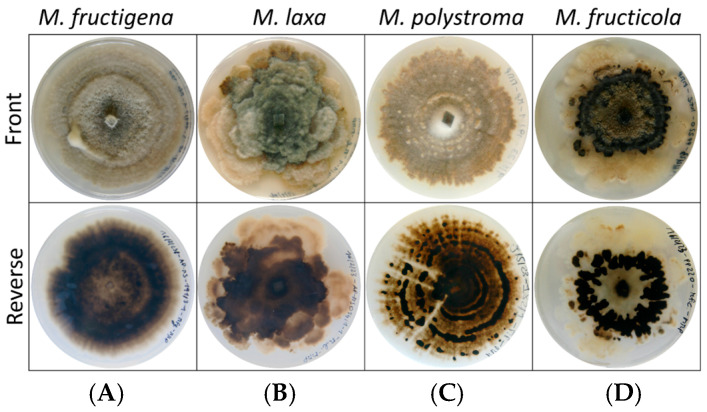
Samples assessed at 27 dpi of (**A**) *M. fructigena* strain 191_3, (**B**) *M. laxa* strain 14_1, (**C**) *M. polystroma* strain 192_1, and (**D**) *M. fructicola* strain PP_220 grown on PDA at 20 °C under a 12:12 h L:D photoperiod. Front and back of colony.

The four *Monilinia* species exhibited distinct morphological characteristics when cultured on PDA. Colonies of *M. fructigena* were characterized by entire or slightly undulate margins and a whitish surface. The reverse side of the colonies appeared dark and frequently displayed concentric rings. *M. polystroma* formed colonies with entire to slightly undulate margins and a grayish coloration. On the reverse side, black stromatal plates arranged in concentric rings were clearly visible, and in some cases, these structures were also observable from the front of the colony. In contrast, colonies of *M. laxa* showed strongly undulated margins and a grayish to brownish coloration. No concentric rings were visible on the reverse side. *M. fructicola* produced colonies with undulated margins and a whitish appearance. Black stromata were evident on the front side of the colony, while the reverse side displayed irregular, undulated stromatal plates that did not originate from the colony center (Figure 2).

**Figure 3:**
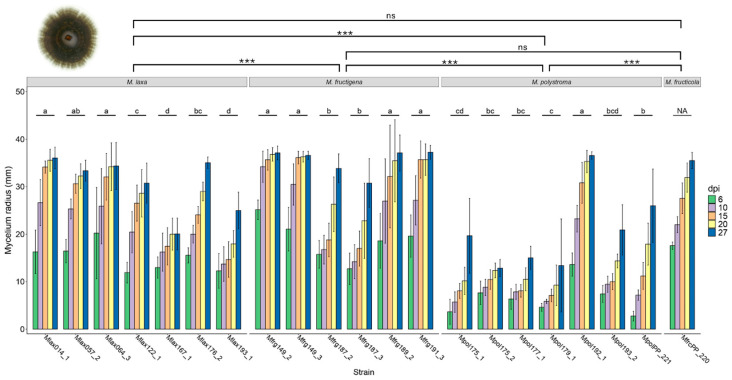
Mean mycelium radius (±SD; *n* = 5) of strains of *M. fructigena*, *M. laxa*, *M. polystroma*, and *M. fructicola* grown on PDA at 20 °C under a 12:12 h L:D photoperiod over 27 days. Different lowercase letters denote significant differences in growth between strains within the same *Monilinia* spp. (*p* < 0.05, *n* = 5). NA means no statistically different values. Asterisks denote significant differences between treatments (^ns^ *p* > 0.05, *** *p* ≤ 0.001).

The mycelial growth rate on PDA varied significantly among the *Monilinia* species (F_3,520_ = 145.57, *p* < 0.001). Over the 27-day observation period, *M. laxa* exhibited a growth rate ranging from 0.64 to 1.45 mm per day. *M. fructigena* grew at a rate of 0.89 to 1.56 mm per day, while *M. fructicola* showed a growth rate between 1.23 and 1.38 mm per day. *M. polystroma* demonstrated a growth rate ranging from 0.22 to 1.39 mm per day (Figure 3).

Significant differences in growth rate were also observed between strains within each species. In *M. laxa*, the effect of strain was significant (F_6,170_ = 41.351, *p* < 0.001). Similarly, growth rate differences among strains of *M. fructigena* (F_5,145_ = 34.987, *p* < 0.001) and *M. polystroma* (F_6,170_ = 67.478, *p* < 0.001) were statistically significant (Figure 3).

### 3.3. Mycelial Growth Rate and Colony Morphology on Apples

**Figure 4:**
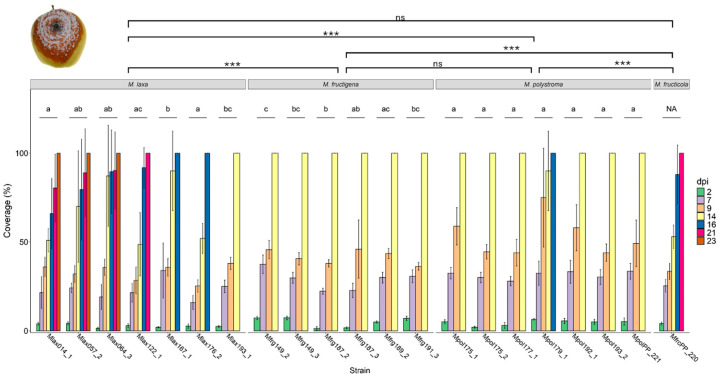
Mean coverage (±SD; *n* = 5) of strains of *M. fructigena*, *M. laxa*, *M. polystroma*, and *M. fructicola* on apples at 20 °C under a 12:12 h L:D photoperiod. Different lowercase letters denote significant differences in growth between strains within the same *Monilinia* spp. (*p* < 0.05, *n* = 5). NA means no statistically significant values. Asterisks denote significant differences between treatments (^ns^ *p* > 0.05, *** *p* ≤ 0.001).

The mycelial growth rate on apples varied considerably among *Monilinia* species and strains (Figure 4). All strains of *M. fructigena* reached full surface coverage within 14 dpi, whereas certain strains of *M. laxa* required up to 23 dpi to achieve full coverage. Statistically, the overall growth rate differed significantly between the *Monilinia* species (F_3,493_ = 33.651, *p* < 0.001). Among them, *M. laxa* displayed the slowest growth, with full coverage reached between 14 and 23 dpi, confirming its significantly reduced colonization rate over the experimental period compared to the other species.

In contrast, both *M. fructigena* and *M. polystroma* exhibited significantly higher growth rates than the tested strain of *M. fructicola*. Most *M. polystroma* strains reached 100% apple surface coverage within 14 dpi. However, one exception was observed for strain Mpol179_1, in which one replicate required 16 dpi to reach complete coverage.

Within species, strain-level differences in growth rate were also significant: In *M. laxa*, growth rate varied significantly between strains (F_6,136_ = 5.892, *p* < 0.001). Similarly, significant variation was found among strains of *M. fructigena* (F_5,116_ = 5.381, *p* < 0.001) and *M. polystroma* (F_6,136_ = 1.918, *p* < 0.001), indicating intraspecific variability in colonization ability on apple fruit (Figure 4).

**Figure 5:**
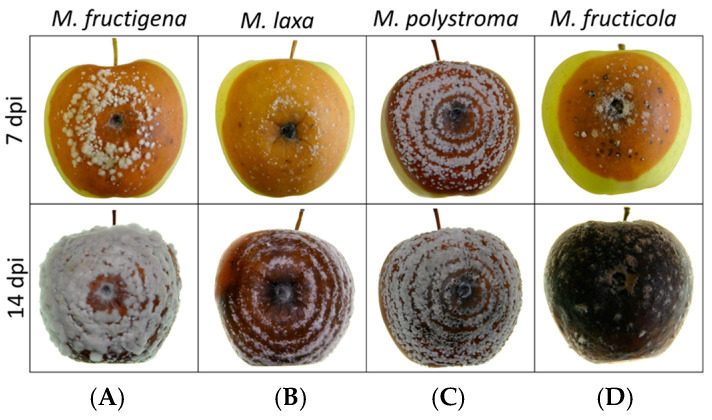
Samples assessed at 7 and 14 dpi of (**A**) *M. fructigena* strain 191_3, (**B**) *M. laxa* strain 14_1, (**C**) *M. polystroma* strain 192_1, and (**D**) *M. fructicola* strain PP_220 grown on apples at 20 °C under a 12:12 h L:D photoperiod.

The colony morphology of the four species on apples was largely similar (Figure 5). In *M. fructigena*, the lesion initially appears light brown, accompanied by dense white mycelial growth forming concentric rings around the inoculation site. As the infection progresses, the center of the lesion darkens, while the mycelium continues to develop a distinct ring-like pattern. In *M. laxa*, the lesion also exhibits a brown discoloration that spreads outward in well-defined concentric rings. However, these rings are more sparsely and less distinctly distributed compared to *M. fructigena*, and white mycelial structures are less prominent. In *M. polystroma*, the lesion is brown with clearly developed concentric rings and abundant white sporulation across the surface. The rings are more closely spaced than in *M. laxa*, and the strong contrast between them gives the colony a distinct patterned appearance. *M. fructicola* also causes a brown lesion, nearly black, 14 dpi. The colony displayed dense, dark sporulation with scattered white stromata, giving it a patchy and irregular appearance. The colony margin is less distinct, and the overall pattern is more diffuse compared to the other species.

### 3.4. Conidia Production and Characterization from PDA, Apple, and TSA

Of the 21 strains tested, only one strain of *M. laxa* sporulated on PDA. In contrast, all strains of *M. fructigena* and *M. fructicola* sporulated on apple between 9 and 21 dpi, whereas *M. laxa* and *M. polystroma* did not sporulate on PDA. On TSA, all *Monilinia* species were capable of sporulation, except for three strains of *M. polystroma*, which showed no sporulation (Table 3).

**Figure 6:**
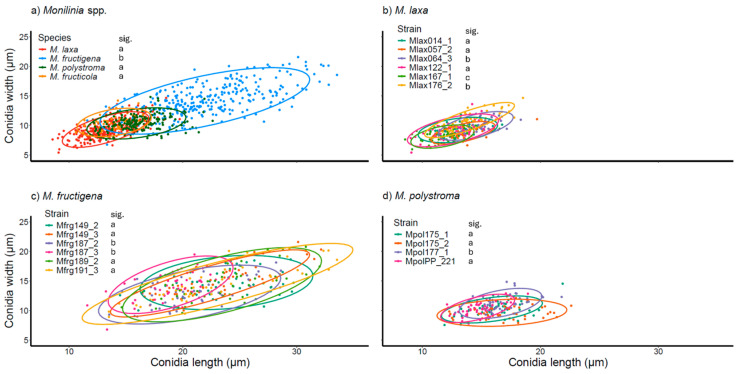
Conidia length and width (*n* = 50) of (**a**) *Monilinia* spp., (**b**) *M. laxa* strains, (**c**) *M. fructigena* strains, and (**d**) *M. polystroma* strains. Ellipses show 95% confidence level. Different lowercase letters indicate significant differences between species or strains (*p* < 0.05).

**Figure 7:**
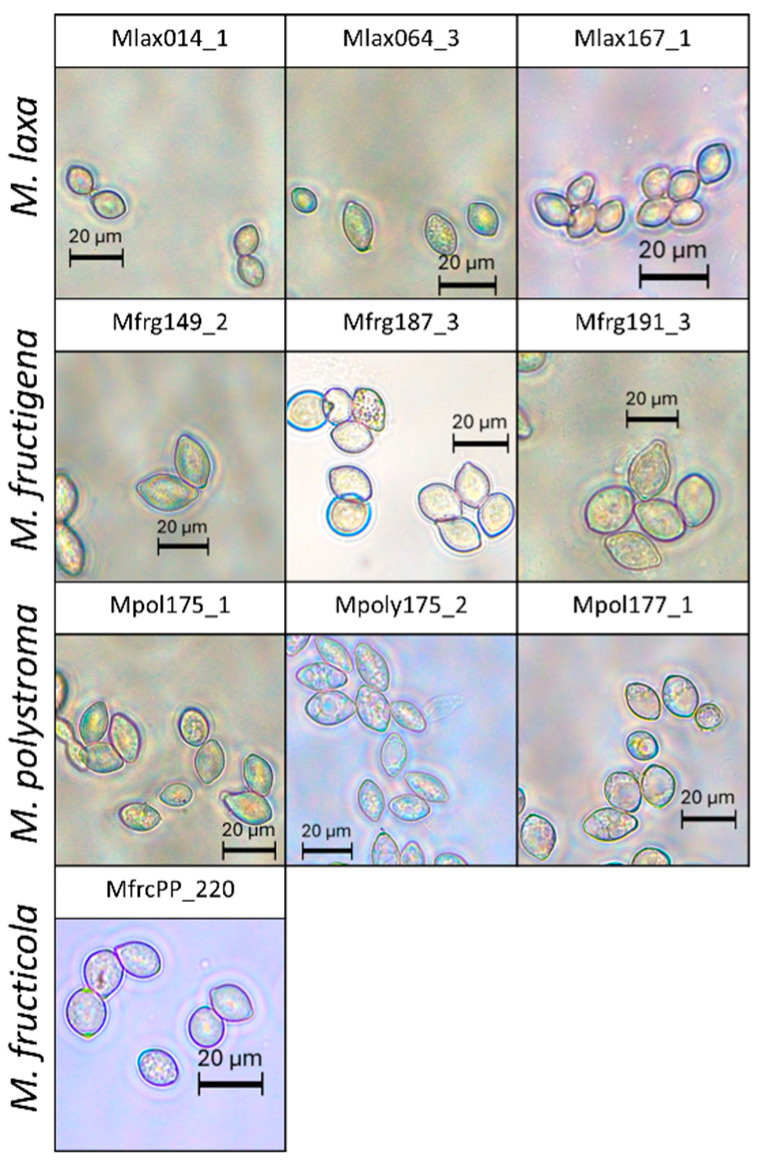
Conidia morphology of selected *Monilinia* strains.

Conidial size differed significantly among *Monilinia* species (F_3,17_ = 59.229, *p* < 0.001). *M. fructigena* produced significantly larger conidia (6.78–21.59 µm × 11.60–33.55 µm) compared to the other species. No significant differences were observed between *M. laxa* (5.44–14.69 µm × 8.55–19.72 µm), *M. fructicola* (8.81–14.24 µm × 10.60–16.75 µm), and *M. polystroma* (7.55–14.85 µm × 11.89–22.62 µm) (Figure 6a). Within species, strain identity also had a significant effect on conidial size (*M. laxa*: F_5_ = 33.73, *p* < 0.001; *M. fructigena*: F_5_ = 8.269, *p* < 0.001; *M. polystroma*: F_3_ = 12.174, *p* < 0.001) (Figure 6b,c). However, due to overlapping 95% confidence intervals, conidial size alone does not allow for reliable discrimination between species or strains (Figure 7).

### 3.5. Fungicide Screening on Apples

**Figure 8:**
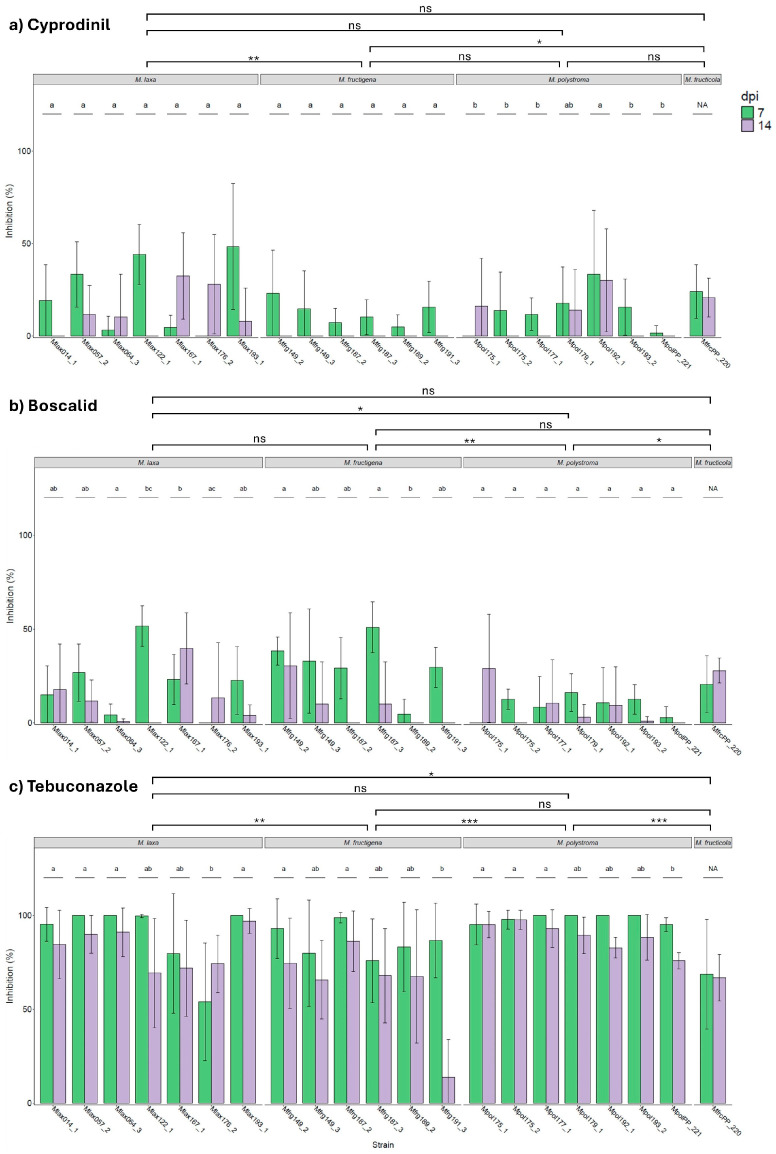
Mean inhibition (±SD; *n* = 5) of *Monilinia* spp. by (**a**) cyprodinil, (**b**) boscalid, and (**c**) tebuconazole on apples at 7 and 14 dpi. Means not sharing any letter are significantly different by the Tukey test (*p* < 0.05). Different lowercase letters denote significant differences in growth between strains within the same *Monilinia* species (*p* < 0.05, *n* = 5). NA means no statistical values. Asterisks denote significant differences between treatments (^ns^ *p* > 0.05, * *p* ≤ 0.05, ** *p* ≤ 0.01, *** *p* ≤ 0.001).

The minimum and maximum inhibition of mycelial growth by cyprodinil on apples ranged from 0 to 89.30% at 7 dpi and from 0 to 60% at 14 dpi. Inhibition was significantly affected by *Monilinia* species (F_3,208_ = 5.142, *p* = 0.002). The highest inhibition rates were observed for *M. laxa* (16.67% at 7 dpi; 12.90% at 14 dpi) and *M. fructicola* (24.03% at 7 dpi; 20.82% at 14 dpi). *M. fructigena* showed the lowest inhibition (12.58% at 7 dpi; 0% at 14 dpi), followed by *M. polystroma* (13.39% at 7 dpi; 8.59% at 14 dpi) (Figure 8a). Among the four species, significant strain-level variation was observed only for *M. polystroma* (F_6,70_ = 3.751, *p* = 0.003).

The minimum and maximum inhibition by boscalid ranged from 0 to 70.97% at 7 dpi and 0 to 66% at 14 dpi. A significant effect of species was detected (F_3,208_ = 5.448, *p* = 0.001), with *M. polystroma* exhibiting significantly lower inhibition (8.98% at 7 dpi; 1.52% at 14 dpi) compared to *M. laxa* (20.42% at 7 dpi; 12.41% at 14 dpi), *M. fructigena* (30.85% at 7 dpi; 8.39% at 14 dpi), and *M. fructicola* (20.57% at 7 dpi; 27.85% at 14 dpi) (Figure 8b). Significant strain effects were found for *M. laxa* (F_6,68_ = 3.431, *p* = 0.005) and *M. fructigena* (F_5,58_ = 5.145, *p* < 0.001).

Tebuconazole exhibited the strongest inhibitory effect, with inhibition ranging from 0 to 100% at both 7 and 14 dpi. Inhibition differed significantly among species (F_3,208_ = 12.381, *p* < 0.001). The highest inhibition was recorded for *M. polystroma* (98.28% at 7 dpi; 88.84% at 14 dpi) and *M. laxa* (89.78% at 7 dpi; 82.53% at 14 dpi). In contrast, *M. fructigena* (86.19% at 7 dpi; 99.33% at 14 dpi) and *M. fructicola* (68.66% at 7 dpi; 66.78% at 14 dpi) showed significantly lower inhibition (Figure 8c). Significant strain effects were detected for *M. laxa* (F_6,68_ = 4.975, *p* < 0.001), *M. fructigena* (F_5,58_ = 3.683, *p* = 0.006), and *M. polystroma* (F_6,68_ = 3.373, *p* = 0.006).

**Figure 9:**
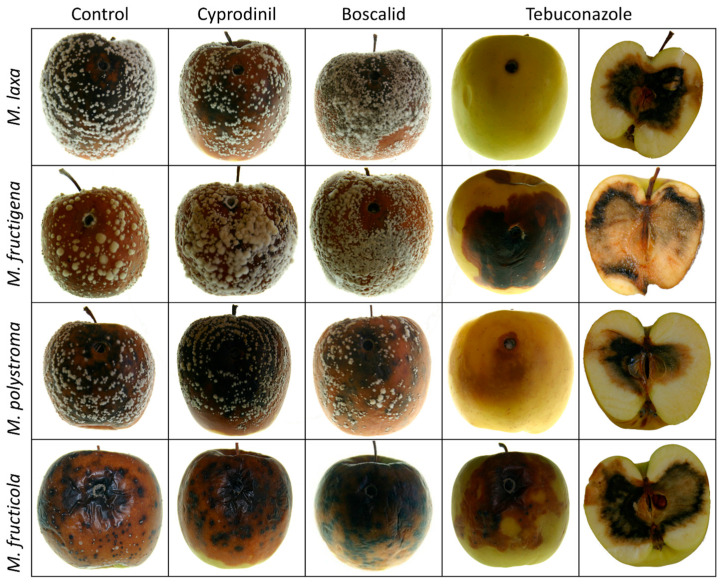
Morphology of *Monilinia* spp. on apples 14 dpi from the control, cyprodinil, boscalid, and tebuconazole treatments. Furthermore, the pulp of apples treated with tebuconazole is shown.

While apples treated with cyprodinil and boscalid showed mycelial growth and brown rot symptoms on the fruit surface, treatment with tebuconazole provided protection on the fruit surface, resulting in fungal spread and symptom development being concentrated in the fruit interior (Figure 9).

## 4. Discussion

This study aimed to characterize four *Monilinia* species (*M. fructigena*, *M. laxa*, *M. polystroma*, and *M. fructicola*) based on morphological traits, phylogenetic identity, growth dynamics, and fungicide sensitivity. In particular, the need for information on *M. polystroma*, a relatively recently described species [22], to address existing knowledge gaps and assess its potential impact on disease management strategies.

Molecular identification using universal primers ITS_5_ and ITS_4_ successfully differentiated among all four *Monilinia* species, supporting the reliability of ITS-based diagnostics for this fungal genus [25]. Sequence homogeneity within species indicated stable ITS regions, with no intraspecific variation observed. Nevertheless, the lack of intraspecific resolution in the ITS region shows that additional molecular markers (e.g., β-tubulin, GAPDH) or whole-genome sequencing is necessary to distinguish different strains based on fungicide sensitivity [33]. The ITS region exhibits generally low variability within *Monilinia* species, yet it remains a useful marker for distinguishing the four described species from others that have not been detected so far in South Tyrol’s alpine orchards [24,34,35]. Morphologically, not all species exhibited distinguishable colony characteristics on PDA; especially, *M. fructigena* and *M. polystroma* displayed entire to slightly undulate margins, highlighting the need for complementary molecular identification.

Colony growth at 20 °C and under short-day conditions revealed significantly lower growth rates compared to the literature values obtained at optimal temperatures [1], reflecting the adaptation to the cooler mountain climates of South Tyrol. Despite these constraints, in vitro growth rate differences among species were consistent with known thermal preferences. *In planta* growth on apples confirmed significant interspecific differences, underscoring the importance of intraspecies diversity in disease progression. Colony morphology and conidial characteristics generally aligned with descriptions from earlier studies [36,37]. Conidia of *M. fructigena* were significantly larger than those of *M. laxa*, *M. fructicola*, and *M. polystroma*, although their 95% confidence intervals overlapped. Therefore, it is not possible to assign *Monilinia* spores to a specific species based on their size.

Sporulation success was highly dependent on the substrate, with TSA outperforming apples in inducing conidial production. However, some *M. polystroma* isolates failed to sporulate on TSA, indicating strain-specific responses to induction media. The results explain the missing sporulation on PDA, which was reported for several experiments to fulfill Koch’s postulates, where mycelial plugs were used [20,38].

The fungicide trials revealed differential sensitivities among the species. The reduced performance of cyprodinil for single strains may indicate the early stages of resistance development, which is consistent with its classification as a high-risk, single-site inhibitor of methionine biosynthesis [13,39,40]. For *M. laxa* isolates, the half maximal effective concentration (EC_50_) values for cyprodinil ranged from 1.11 to 0.03 mg L^−1^, and for *M. fructicola*, from 0.29 to 0.06 mg L^−1^ [39]. The observed cyprodinil inhabitation at a dose of 250 mg L^−1^ ranged from 0 to 89.30%, which indicates a high strain and species-specific variation.

Boscalid, belonging to the SDHI class (FRAC Group 7) and acting by the inhibition of Complex II of mitochondrial respiration [13,14,15], showed moderate efficacy. Resistance arises from mutations in the Sdh gene complex (SDHA–D subunits) and overexpression of efflux transporters, reducing intracellular fungicide concentrations [15,27]. For *M. fructicola* isolates, the EC_50_ values for boscalid range from 0.42 to 11.94 mg L^−1^ [27]. At a boscalid concentration of 200 mg L^−1^, inhibition across *Monilinia* species ranged from 0 to 70.97%, again highlighting a substantial variation between strains and species.

Tebuconazole, a DMI fungicide, showed the highest efficacy across species, overall, close to the skin of treated apples. This aligns with its systemic activity and broad spectrum of effectiveness [16,17]. For *M. laxa* isolates, the EC_50_ values ranged from 0.42 to 0.02 mg L^−1^, and for *M. fructicola*, from 0.37 to 0.13 mg L^−1^ [41]. The observed tebuconazole inhibition at a dose of 125 mg L^−1^ ranged from 0 to 100%, which indicates a high strain and species-specific variation, as it was observed for the other fungicides. Overall high tolerances in *M. laxa* and *M. polystroma* indicate the presence of resistant strains.

The findings suggest that fungicide application strategies should account for species-specific sensitivities. Reduced efficacy of cyprodinil and boscalid could stem from repeated applications and selection pressure [1]. Indeed, despite operating through a distinct mode of action, the observed partial tolerance to both cyprodinil and boscalid in some strains could hint at emerging cross-resistance mechanisms, potentially linked to multidrug efflux transporters or stress response pathways [5]. Rotational and integrated fungicide programs, as recommended by FRAC, are necessary to delay resistance onset [13]. Post-harvest brown rot management is particularly challenging in Europe due to the absence of authorized chemical treatments [10]. This makes pre-harvest fungicide efficacy critical for disease suppression.

In the alpine region of South Tyrol, the climate and cultivation practices may favor *M. laxa* dominance, but the presence of *M. polystroma* and *M. fructicola*, species with distinct thermal preferences, could complicate disease dynamics, especially under climate change scenarios. Studies from central and southern Europe have shown varying dominance of *Monilinia* species and differences in fungicide sensitivity patterns [41,42,43]. Comparing our findings with those studies highlights the importance of region-specific monitoring programs and suggests that climatic conditions and orchard practices significantly shape pathogen populations.

## 5. Conclusions

In conclusion, ITS sequencing with primers ITS_5_ and ITS_4_ successfully differentiated all species, with no intraspecific variation observed. Colony morphology and conidial size showed species-specific trends, but overlapping ranges prevent reliable identification based on morphology alone. All species sporulated on TSA, while PDA was less suitable. Some *M. polystroma* isolates failed to sporulate on TSA, indicating strain-specific differences. Further studies should focus on identifying compounds that are responsible for the induction of sporulation. Fungicide trials at field concentration revealed significant variation in sensitivity. The variability across and within species emphasizes that the occurrence of species and strains can substantially affect fungicide efficacy. This underlines the importance of studies addressing strain-specific differences in fungicide sensitivity and resistance, especially in fruit growing regions such as South Tyrol’s alpine orchards.

## Figures and Tables

**Table 1:** Descriptive table of 21 *Monilinia* strains: assigned species, strain ID, host fruit from which they were collected, and the respective sampling site in German and Italian.

Species	Strain ID	Host Fruit	Sampling Sites *
*M. laxa*	Mlax014_1	Plum	Jenesien/San Genesio
	Mlax057_2	Peach	Partschins/Parcines
	Mlax064_3	Cherry	Allitz/Lasa
	Mlax122_1	Cherry	Ritten/Renon
	Mlax167_1	Apricot	Kollmann/Colma
	Mlax176_2	Peach	Piglon/Piccolungo
	Mlax193_1	Quince	Kaltern/Caldaro
*M. fructigena*	Mfrg149_2	Plum	Kollmann/Colma
	Mfrg149_3	Plum	Kollmann/Colma
	Mfrg187_2	Apple	Terlan/Terlano
	Mfrg187_3	Apple	Terlan/Terlano
	Mfrg189_2	Almond	Piglon/Piccolungo
	Mfrg191_3	Almond	Piglon/Piccolungo
*M. polystroma*	Mpol175_1	Cherry	Laimburg
	Mpol175_2	Cherry	Laimburg
	Mpol177_1	Almond	Piglon/Piccolungo
	Mpol179_1	Almond	Piglon/Piccolungo
	Mpol192_1	Quince	Kaltern/Caldaro
	Mpol193_2	Quince	Kaltern/Caldaro
	MpolPP_221	Apple	Japan (CBS 102686)
*M. fructicola*	MfrcPP_220	Cherry	Bari, Italy (CBS 144849)

* Sampling site names are provided in German and Italian.

**Table 2:** Fungicides used in laboratory bioassays, including active ingredient (AI), trade name, manufacturer/distributor, formulation, and applied dose.

Active Ingredient	Trade Name	Manufacturer/Distributor	Composition (AI)	Applied Dose (AI)
Boscalid	Cantus^®^	BASF Italia S.p.A. (Cesano Maderno, Italy)	500 g kg^−1^	200 mg L^−1^
Cyprodinil	Chorus^®^	Syngenta Italia S.p.A. (Milan, Italy)	500 g kg^−1^	250 mg L^−1^
Tebuconazole	Folicur^®^ WG	Bayer CropScience S.r.l. (Milan, Italy)	250 g kg^−1^	125 mg L^−1^

**Table 3:** Sporulation capability of *Monilinia* species on PDA, apple, and TSA of the 21 strains and the respective days required for sporulation to happen (“X” = sporulation occurred, “-” = not sporulating).

Species	Isolate	Sporulation					
		PDA	dpi	Apple	dpi	TSA	dpi
*M. laxa*	Mlax014_1	-	-	-	-	X	6
	Mlax057_2	-	-	-	-	X	12
	Mlax064_3	-	-	-	-	X	6
	Mlax122_1	-	-	-	-	X	6
	Mlax167_1	X	12	-	-	X	12
	Mlax176_2	-	-	-	-	X	6
	Mlax193_1	-	-	-	-	X	12
*M. fructigena*	Mfrg149_2	-	-	X	9	X	6
	Mfrg149_3	-	-	X	9	X	7
	Mfrg187_2	-	-	X	14	X	7
	Mfrg187_3	-	-	X	9	X	6
	Mfrg189_2	-	-	X	14	X	7
	Mfrg191_3	-	-	X	21	X	6
*M. polystroma*	Mpol175_1	-	-	-	-	X	6
	Mpol175_2	-	-	-	-	X	12
	Mpol177_1	-	-	-	-	X	12
	Mpol179_1	-	-	-	-	-	-
	Mpol192_1	-	-	-	-	-	-
	Mpol193_2	-	-	-	-	-	-
	MpolPP_221	-	-	-	-	X	6
*M. fructicola*	MfrcPP_220	-	-	X	16	X	6

## Data Availability

The ITS sequences are identical to those already available in the NCBI GenBank database (accession numbers: MH864497, MH862738, MH855219, MH863200). The corresponding raw data and raw sequencing files have been deposited in Zenodo and are available at: https://doi.org/10.5281/zenodo.17167654.
